# Effect of long-acting injectable paliperidone 3 monthly and aripiprazol 1 monthly on hospitalization rate in a first-episode psychosis

**DOI:** 10.1192/j.eurpsy.2021.2146

**Published:** 2021-08-13

**Authors:** P. Gil Lopez, J.M. Rodriguez Sanchez, L. Garcia Fernandez, V. Sanchez Estevez

**Affiliations:** Early Intervention Service For First Psychotic Episodes “lehenak”, Mental Health Network of Bizkaia (RSMB). Basque Health System. Osakidetza, Bilbao, Spain

**Keywords:** first-episode psychosis, Relapse prevention, long-acting aripiprazol, long-acting paliperidone

## Abstract

**Introduction:**

Long-acting injectable antipsychotics (LAIs) can reduce relapse and hospitalization risk but they are not widely used in first psychotic episode (FEP) patientes.

**Objectives:**

To examine the effcacy of two of the most used second generation LAI antipsychotics (paliperione 3 monthly and aripiprazol 1 monthly) to reduce hospitalization rates.

**Methods:**

We evaluated in a naturalistic study a sample of patients (n=277) with a FEP. We carried out a mirror-design study to compare the number of hospitalizations and days in hospital before and after the introduction of LAI paliperidone (3 monthly) or LAI aripiprazol. In our Early Intervention Services (Lehenak) antipsychotic treatment is not protocolized and is established for each patient according to the psychiatrist criteria.

**Results:**

We review the oucome of 277 FEP treated in our Early Intervention Service “Lehenak” with LAI paliperidone 3 monthly (n=156) or LAI Aripiprazol (n=121)

**Table:** 

	Pre LAI Mirror Period	Post LAI Mirror Period	Within group comparisons (paired t-test) t p
Aripiprazol LAI number of Hospitalizations (mean, standard deviation)	2.31 (1.72)	0.73 (1.23)	17.4 (<0.001)
Paliperidone LAI 3 monthly number of Hospitalizations number	0,68 (0.93)	0.15 (0.47)	4.62 (<0.001)
Aripiprazol LAI Days in Hospital	30.26 (33.52)	17.02 (38.19)	2.93 (0.004)
Paliperidone LAI 3 monthly Days in hospital	12.63 (24.23)	3.40 (14.18))	7.5 (<0.001)

**Conclusions:**

Both LAI paliperidone 3 monthly and LAI aripiprazol had a postive impact on hospitalIzation rate, decreasing them significantly after their introduction. These data also support a more extensive use of LAI paliperidone 3 monthly in FEP.

**Disclosure:**

Presenting author has received honouraria for lectures or advisory boards from Janssen, Otsuka, Lundbeck and Angelini in the last five years

